# Laparoscopic Repair of a Femoral Hernia Involving the Appendix in a Patient With a History of Extracorporeal Membrane Oxygenation After Myocardial Infarction

**DOI:** 10.7759/cureus.86930

**Published:** 2025-06-28

**Authors:** Toru Kusano, Touma Mijin, Yuki Koga, Chusei Ryu, Toshikazu Matsuo

**Affiliations:** 1 Gastroenterological Surgery, Omura Municipal Hospital, Omura, JPN

**Keywords:** de garengeot hernia, ecmo, femoral hernia, laparoscopic appendectomy, laparoscopic trans-abdominal pre-peritoneal repair

## Abstract

We present the case of a patient who underwent laparoscopic appendectomy and transabdominal preperitoneal repair for a femoral hernia involving the appendix after myocardial infarction and required veno-arterial extracorporeal membrane oxygenation (VA-ECMO). We admitted a 61-year-old man who was experiencing swelling and pain in his right groin. His medical history included a myocardial infarction three years prior, for which he required VA-ECMO using the cutdown technique in our hospital. Abdominal computed tomography revealed a right femoral hernia involving the appendix, and the patient underwent laparoscopic surgery. During laparoscopy, the appendix, which had disengaged from the right femoral hernia, was facilitated by visualization. The appendix exhibited mild inflammation, and no abscess was found in the lumen of the right femoral hernia; therefore, the patient underwent laparoscopic appendectomy and transabdominal preperitoneal repair. In this patient with a femoral hernia involving the appendix, laparoscopic surgery was useful in the identification of the appendix, although the incarcerated appendix had disengaged from the femoral hernia at the time of surgery. Because it is unclear whether VA-ECMO using the cutdown technique is associated with femoral hernias, further case reports similar to ours would be valuable.

## Introduction

First reported in 1731, a femoral hernia involving the appendix, also termed as de Garengeot hernia, is an extremely rare presentation [1]. Wakele et al. reported that appendiceal incarceration was observed in only three of 610 (0.49%) patients with a femoral hernia [2]. Here, we describe the case of a patient with a history of myocardial infarction requiring veno-arterial extracorporeal membrane oxygenation (VA-ECMO), who underwent laparoscopic repair of a femoral hernia involving the appendix three years later.

## Case presentation

**Figure 1:**
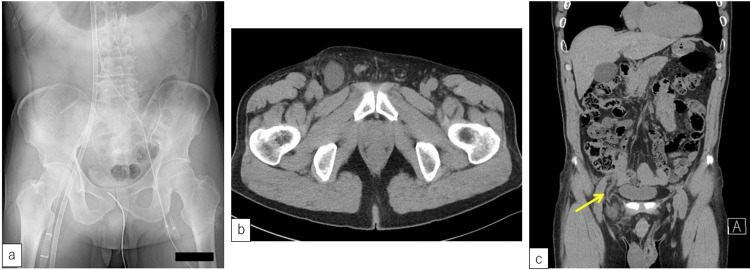
Abdominal X-ray during VA-ECMO and abdominal computed tomography of the femoral hernia involving the appendix. a: VA-ECMO blood inflow cannula is inserted through the right femoral artery, and the blood outflow cannula is inserted through the right femoral vein, and an intra-aortic balloon pumping catheter is inserted through the left femoral artery; b: despite the presence of fluid retention, no signs of perforation are observed; c: the appendix is observed in the right femoral hernia (arrow). (b: axial section; c: coronal section). VA-ECMO: veno-arterial extracorporeal membrane oxygenation

A 61-year-old man was admitted to the hospital with swelling and pain in the right groin. Before the admission, his Eastern Cooperative Oncology Group (ECOG) performance status was grade 0, and he had maintained his weight for over 10 years. He had no notable sports history, and he worked in agricultural fields. He drank alcohol occasionally and smoked three cigarettes a day for 40 years. However, he had a three-year history of myocardial infarction requiring VA-ECMO (Figure 1a). During VA-ECMO, a cannula was inserted percutaneously into the right femoral artery and vein and then removed using a cutdown technique. There were no access site complications, including postoperative hematoma, vessel dissection, and pseudoaneurysm.

When he was admitted to the hospital, he did not have peritonitis, and the pain was localized to the right groin area. Although he had no fever and his blood examination results showed no increased inflammation, abdominal computed tomography revealed a right femoral hernia involving the appendix (Figures 1b, 1c). We diagnosed a de Garengeot hernia, and laparoscopic surgery was performed after obtaining patient consent.

**Figure 2:**
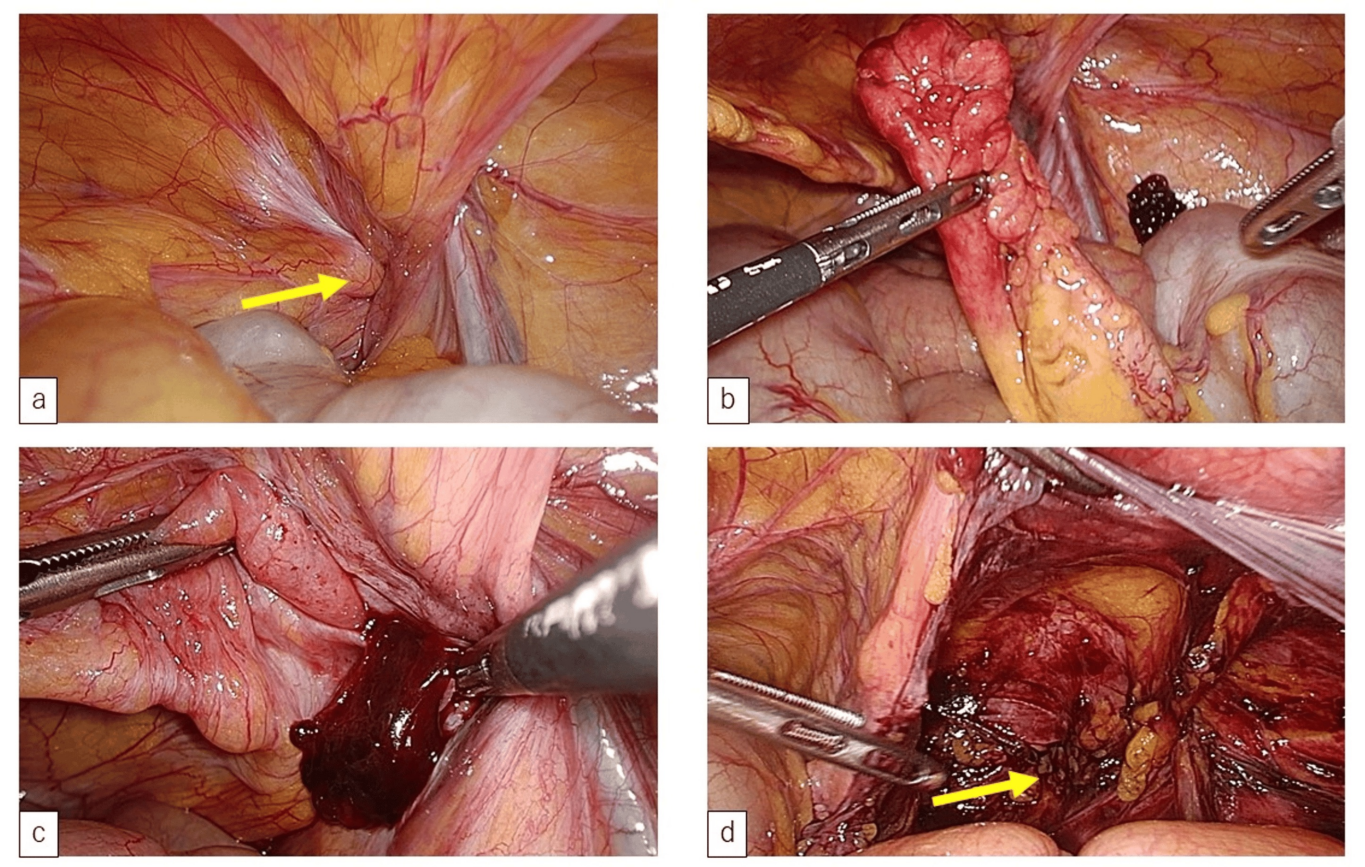
Laparoscopic findings of the appendix and right femoral hernia. a: the appendix is disengaged from the right femoral hernia (arrow); b: the appendix is easily identifiable, and changes following the impaction of the appendix in the femoral hernia are shown; c: a hematoma is observed in the femoral hernia; d: the peritoneum is incised and the femoral canal is identified (arrow).

Briefly, a three-port laparoscopy was performed under general anesthesia, with the patient placed in a supine position. During laparoscopy, the appendix, which had disengaged from the right femoral hernia, was easily identifiable (Figures 2a, 2b). Laparoscopic appendectomy with transabdominal preperitoneal repair (TAPP) was performed because of the mildly inflamed appendix and the absence of an abscess in the lumen of the right femoral hernia (Figures 2c, 2d). The procedure duration was 125 min, and bleeding was limited. The postoperative course was uneventful, and the patient was discharged on postoperative day three. The pathologic examination of the resected appendix led to the diagnosis of fibrous occlusion and phlegmonous appendicitis.

## Discussion

Femoral hernias, which account for 4%-8% of all inguinal hernias, are more common in middle-aged and older women [3]. In a femoral hernia, the femoral ring, i.e., the hernia opening, is narrow, and the femoral vascular sheath and hiatal ligament tightly grip the hernia neck. Therefore, approximately 70% of all femoral hernias become incarcerated [4,5].

A femoral hernia involving the appendix is called a de Garengeot hernia, and it was first reported in 1731 [1]. It has been reported that appendiceal incarceration occurs in three out of 610 (0.49%) cases of femoral hernia [2]. Hernias involving the appendix can originate not only from the femoral canal but also from the inguinal canal, which is also known as Amyand's hernia, with a reported incidence of 0.97% (12/1232) of all inguinal hernias [2,6].

Incarceration of the appendix in patients with femoral hernia might be precipitated by a large cecum, a cecum positioned low in the pelvis, or abnormal intestinal rotation [7-9]. Abdominal ultrasound and computed tomography are useful for diagnosing femoral hernia, which can be observed as a bulge caudal to the inguinal ligament and compression of the femoral vein from the medial side [10].

A femoral hernia with an incarcerated appendix is often complicated by appendicitis and accompanied by symptoms of incarceration, such as a painful mass or swelling in the groin. However, as inflammation progresses, it can further tighten the hernia opening, preventing the spread of inflammation to the abdominal cavity. As a result, symptoms of peritoneal inflammation are rare in cases of femoral hernia with an incarcerated appendix. Instead, inflammation tends to spread from the groin to the subcutaneous soft tissue and skin of the thigh [11,12].

Our patient did not have the typical symptoms of peritonitis or appendicitis. The appendix, which had disengaged from the right femoral hernia at the time of surgery, was easily identifiable using laparoscopy. The definitive treatment for de Garengeot hernia is a surgical appendectomy combined with hernia repair [13]. However, it has been suggested that if the appendix is ​​normal, appendectomy is unnecessary [4,14]. In our case, laparoscopic appendectomy and TAPP were performed due to the mild inflammation of the appendix and the absence of an abscess in the lumen of the right femoral hernia, leading to a successful outcome.

Importantly, the patient had a history of myocardial infarction requiring VA-ECMO, which was performed three years earlier. During VA-ECMO, a cannula was inserted percutaneously into the right femoral artery and vein and later removed using a cutdown technique. Although there are reports that compare percutaneous versus cutdown access for endovascular abdominal aortic repair and thoracic endovascular aortic repair, no study has reported cutdown access as a risk factor for femoral hernia in long-term outcomes [15,16]. The cutdown technique requires taping and dissection of the femoral artery and vein near the inguinal ligament and may also require dissection of the inguinal ligaments, which may weaken the tissue around the femoral canal and predispose to femoral hernias. In our case, although there is no mention in the operation record about the dissection of the inguinal ligaments, the surrounding tissues of the femoral canal may have been weakened. It remains unclear whether VA-ECMO using the cutdown technique contributed to the incarceration of the appendix in the femoral hernia in our patient, but this possibility should be considered in future reports.

## Conclusions

Although the incarcerated appendix had disengaged from the femoral hernia at the time of laparoscopic surgery, our treatment approach was useful in facilitating visualization of the appendix. Future studies should consider whether VA-ECMO using the cutdown technique is associated with femoral hernia, and further case reports similar to ours would be valuable.
